# Discovery of Predictors of *Mycoplasma hyopneumoniae* Vaccine Response Efficiency in Pigs: 16S rRNA Gene Fecal Microbiota Analysis

**DOI:** 10.3390/microorganisms8081151

**Published:** 2020-07-29

**Authors:** Peris M. Munyaka, Fany Blanc, Jordi Estellé, Gaëtan Lemonnier, Jean-Jacques Leplat, Marie-Noëlle Rossignol, Déborah Jardet, Graham Plastow, Yvon Billon, Benjamin P. Willing, Claire Rogel-Gaillard

**Affiliations:** 1 Université Paris-Saclay, INRAE, AgroParisTech, GABI, 78350 Jouy-en-Josas, France; munyakam@ualberta.ca (P.M.M.); fany.blanc@inrae.fr (F.B.); jordi.estelle@inrae.fr (J.E.); gaetan.lemonnier@inrae.fr (G.L.); jean-jacques.leplat@inrae.fr (J.-J.L.); marie-noelle.rossignol@inrae.fr (M.-N.R.); deborah.jardet@inrae.fr (D.J.); 2 Department of Agricultural, Food and Nutritional Sciences, University of Alberta, Edmonton, AB T6G 2R3, Canada; plastow@ualberta.ca (G.P.); willing@ualberta.ca (B.P.W.); 3 Livestock Gentec, University of Alberta, Edmonton, AB T6G 2R3, Canada; 4 INRAE, GenESI, 17700 Surgères, France; yvon.billon@inrae.fr

**Keywords:** vaccination, microbiota, pig, prediction, *Mycoplasma hyopneumoniae*

## Abstract

The gut microbiota comprises a large and diverse community of bacteria that play a significant role in swine health. Indeed, there is a tight association between the enteric immune system and the overall composition and richness of the microbiota, which is key in the induction, training and function of the host immunity, and may therefore, influence the immune response to vaccination. Using vaccination against *Mycoplasma hyopneumoniae* (*M. hyo*) as a model, we investigated the potential of early-life gut microbiota in predicting vaccine response and explored the post-vaccination dynamics of fecal microbiota at later time points. At 28 days of age (0 days post-vaccination; dpv), healthy piglets were vaccinated, and a booster vaccine was administered at 21 dpv. Blood samples were collected at 0, 21, 28, 35, and 118 dpv to measure *M. hyo*-specific IgG levels. Fecal samples for 16S rRNA gene amplicon sequencing were collected at 0, 21, 35, and 118 dpv. The results showed variability in antibody response among individual pigs, whilst pre-vaccination operational taxonomic units (OTUs) primarily belonging to *Prevotella*, [*Prevotella*], *Anaerovibrio*, and *Sutterella* appeared to best-predict vaccine response. Microbiota composition did not differ between the vaccinated and non-vaccinated pigs at post-vaccination time points, but the time effect was significant irrespective of the animals’ vaccination status. Our study provides insight into the role of pre-vaccination gut microbiota composition in vaccine response and emphasizes the importance of studies on full metagenomes and microbial metabolites aimed at deciphering the role of specific bacteria and bacterial genes in the modulation of vaccine response.

## 1. Introduction

Early-life gut microbiota diversity and composition have been identified as key players for health and disease in both animals and humans [1,2,3,4]. Among the many functions ascribed to the intestinal microbiota are the roles in shaping local mucosal and systemic immunity, and in protecting the host from pathogen expansion and invasion [5,6]. This phenomenon is well studied using germ-free animals, which lack microbial exposures and hence, are associated with several immunologic abnormalities [7,8]. Therefore, since the intestinal microbiota can alter the immune response to potential pathogens, by extension, it may influence how individuals respond to vaccinations [9]. Also, for reasons that are poorly understood, individual response to vaccination is highly variable among populations [10], and research suggests that variations in microbial communities, due to environmental, nutritional, and hygiene conditions, could partly explain the observed heterogeneity in vaccine responses [11].

The immunological basis for how the microbiome may influence vaccination has been a subject of exploration for several years [12,13], and more recent studies have expanded upon this topic [10,14,15,16]. In this context, a more diverse intestinal microbiota has been shown to be favorable for a good immune response to oral vaccines against intestinal pathogens [12]. Also, toll-like receptor 5 (TLR5) sensing of flagellin has been associated with adjuvant activities in response to inactivated influenza vaccine and promotion of plasma cell differentiation in mice [17], whilst the composition of stool microbiota in infants has been shown to correlate with vaccine-specific immune responses [18,19]. Furthermore, antibiotic exposure in infant mice has been associated with impaired antibody response to five widely used human vaccines [20]. Moreover, a study investigating specific pathogen-free layer chickens suggested that shifts in gut microbiota composition may influence both cell- and antibody-mediated immune responses, following vaccination against influenza viruses [21]. These studies support the immunological capacity of the microbiota, or its metabolites, to act as ‘endogenous vaccine adjuvants’ [14], potentially capable of being harnessed to amplify adaptive immune responses to specific pathogens.

We recently investigated the potential contribution of early gut microbiota in the modulation of the immune response, particularly in the context of vaccine response in pigs, using *M. hyo* vaccine as an exemplifier [22]. Our results highlighted the potential for pre-vaccination early-life microbiota to predict vaccine response to *M. hyo* in pigs. Therefore, the aim of this study was to investigate whether early-life pre-vaccination gut microbiota could also predict immune response to *M. hyo* vaccination, using a larger population of pigs from a different genetic background and environment. We also explored possible associations between the presence and relative abundance of distinct bacterial communities in the swine gut at different time points, and vaccine response. 

## 2. Materials and Methods

### 2.1. Ethical Considerations

All animal experiments were carried out in accordance with European Guidelines for the Care and Use of Animals for Research Purposes. The animal protocol was assessed by the local ethics committee in Poitou Charentes and assigned the approval number APAFIS#4295-2016022615583351v4 (11 October 2016) by the French Ministry of Research. 

### 2.2. Animal Design, Vaccination and Sampling Protocol

**Figure 1:**
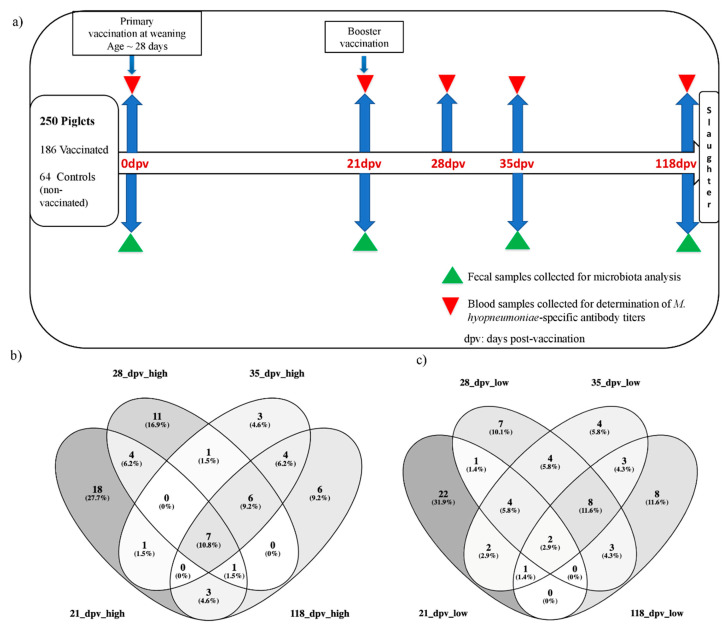
Study design, vaccination, fecal and blood sampling protocol, and groups of pigs classified as High or Low responders. (**a**) Protocol design and sampling. (**b**) Venn diagram showing shared and unshared groups of pigs classified as High responders based on antibody titers determined at different post-vaccination time points, (**c**) Venn diagram showing shared and unshared groups of pigs classified as Low responders based on antibody titers determined at different post-vaccination time points. Dpv; days post-vaccination.

The animal design and vaccination protocol are as described by Blanc et al. [23]. Figure 1a shows a summary of the vaccination and sampling protocol. Briefly, a total of 278 healthy Large White piglets from 48 different litters (145 uncastrated males and 133 females) were produced in five batches and raised without antibiotic treatment at Le Magneraud experimental farm [24]. In each litter, animals were selected with attention to sex ratio and weight as measured seven days before vaccination, and four to five piglets were vaccinated against *M. hyo* (*n* = 203), whereas one or two animals were non vaccinated (*n* = 75). In this context, the first dose of vaccine was given at 0-day post-vaccination (dpv), corresponding to the weaning date (at 28 days of age on average; from 24 to 31 days of age), and a booster vaccine was administered at 21 dpv. Piglets from the same litter were kept together until weaning. During the post-weaning period (until 70 days of age), groups of piglets from two litters were housed together in separate pens, whereas during the growing period (from 70 days of age to slaughtering), the pig groups were reorganized further in separate pens and where possible, pigs from the same litter were kept together.

Pigs that were not sampled at least until 35 dpv for reasons independent of the vaccination protocol were removed from the study. Also, four vaccinated pigs that did not show any response to vaccination were removed from the study as we could not ascertain whether they were non-responders, or it was due to technical failure of the vaccination injection. Therefore, the final dataset comprised 186 piglets vaccinated against *M. hyo* and 64 control non-vaccinated piglets. Pigs were monitored daily throughout the experimental period. Peripheral blood (jugular vein) was sampled using dry tubes for serum preparation at 0, 21, 28, 35, and 118 dpv. At 0, 21, 35, and 118 dpv, fecal samples for microbiota profiling were collected directly from rectal *ampulla*, snap-frozen in liquid nitrogen and transferred to −80 °C freezer for storage until use for microbial DNA extraction.

### 2.3. Determination of Antibody Response against M. hyo and Selection of High and Low Responder Pigs

The levels of *M. hyo*-specific Abs were measured using a commercial IDEXX enzyme-linked immunosorbent assay (ELISA) kit (IDEXX Europe B.V., Hoofddorp, The Netherlands), following the manufacturer’s instructions. As reported by Blanc et al. [23], Ab levels were calculated by dividing the absorbance of the samples (S, corrected by subtraction of the mean negative control absorbance) by the mean absorbance of the positive control (P, corrected by subtraction of the mean negative control absorbance), which resulted in an S/P value. Inter-individual variations in vaccine response against *M. hyo* allowed us to select extreme groups based on antibody levels determined at 21, 28, 35, and 118 dpv, using the S/P values obtained from the vaccinated pigs (*n* = 186). In this context, High and Low responders corresponded to pigs with an Ab response higher than the mean +1 standard deviation (SD) or less than the mean −1 SD, respectively. They were, therefore, used in the determination of the differentially abundant operational taxonomic units (OTU)s or genera, or overall differences in microbiota composition, before vaccination (0 dpv). Figure 1b,c shows a representation of the High and Low responder pigs selected at each post-vaccination time point, as well as the shared and unshared individuals between different time points. On the one hand, only seven pigs were consistently classified as High at 21, 28, 35, and 118 dpv, whereas six other pigs were consistently classified as High responders at 28, 35, and 118 dpv (Figure 1b). On the other hand, only two pigs were consistently classified as Low responders at 21, 28, 35, and 118 dpv, whereas eight pigs were consistently classified as Low responders at 28, 35, and 118 dpv (Figure 1c). This was primarily because of the shifting of antibody levels in individual pigs from High to the middle or from Low to the middle at different time points. Therefore, due to the low consistency in the groups of pigs classified as High or Low responders at different post-vaccination time points, the pre-vaccination microbiota comparisons/analyses between the High vs. Low responder pigs were done based on each dpv classification, independently. Table S1 shows the pigs that were selected as High or Low responders together with the S/P levels, at different post-vaccination time points.

### 2.4. Fecal DNA Preparation

Total DNA was extracted from all fecal samples collected at postnatal day (PND) 28, 49, 63 and 146 (corresponding to 0, 21, 35, and 118 dpv), using a modified version of the protocol by Godon and colleagues [25] adapted to the Chemagic STAR nucleic acid workstation (Hamilton, Perkin Elmer, Reno, NV, USA) as described previously [26]. Starting from 200 mg of frozen fecal matter, each sample was incubated at 70 °C for 1 h in a mixture of 250 μL of guanidine thiocyanate buffer (4 M guanidine thiocyanate—0.1 M Tris (pH 7.5)), 40 μL of 10% N-lauroyl sarcosine—0.1 M phosphate buffer (pH 8.0), and 500 μL of 5% N-lauroyl sarcosine. A 750 μL volume of 0.1 mm-diameter silica beads (Sigma-Aldrich, Saint-Louis, MO, USA, Germany) was added to samples, and the tubes were shaken for 10 min at 25 agitations per second in an MM301 Mixer Mill (Retsch, Haan, Germany). The samples were subsequently centrifuged at 14,000 rpm and 4 °C for 5 min, and supernatants were collected into new tubes. Then samples were added with 30 μL of Proteinase K (chemagic STAR DNA BTS Kit, Perkin Elmer, USA) and incubated 10 min at 250 rpm and 70 °C in a MultiTherm Vortexer (Benchmark Scientific, Sayreville, NJ, USA). After a final 5 min heating step at 95 °C for enzyme inactivation, samples were again centrifuged at 14,000 rpm and 4 °C for 5 min, and supernatants were transferred into deep-well plates for further extraction using the chemagic STAR DNA BTS Kit (Perkin Elmer, Wellesley, MA, USA), following the manufacturer’s instructions (starting after the Protease K incubation step). DNA purity and concentration were measured using a NanoDrop spectrophotometer (NanoDrop Technologies, Wilmington, DE, USA).

### 2.5. 16S rRNA Gene Sequencing and Bioinformatics Analyses

Amplicon libraries from the V3–V4 region of the 16S rRNA gene were constructed and amplified using the PCR1F_343 (5′-CTTTCCCTACACGACGCTCTTCCGATCTACGGRAGGCAGCAG-3′) and PCR1R_784 (5′-GGAGTTCAGACGTGTGCTCTTCCGATCTTACCAGGGTATCTAATCCT-3′) primers following the Illumina 16S metagenomic sequencing library preparation protocol. Paired-end sequencing of the pooled library was performed on an Illumina MiSeq platform (Illumina Inc., San Diego, CA, USA) using the Miseq Reagent kit v3 (2 × 300 cycles, Illumina Inc., San Diego, CA, USA). Downstream analysis was performed using QIIME 1.9.1 (Quantitative Insight into Microbial Ecology, city, state, country) [27] as described previously [28]. Briefly, the multiple_join_paired_ends.py function in QIIME was used to merge the forward and reverse reads contained in the fastq files of each sample. Next, the multiple_split_libraries_fastq.py command was used to demultiplex and filter the fastq sequence data, and OTUs were identified, using the pick_open_reference_otus.py function with a subsampled percentage of 10% (*s * =  0.1), using the GreenGenes database (v. 13.8) clustered at 97% identity. Subsequently, chimera detection was carried out in QIIME1, using BLAST. After all the quality checks and filtering, the samples that did not satisfy the quality filters were discarded. The final dataset was obtained after filtering out OTUs, representing less than 0.005% of the total number of annotated reads [29]. Samples from animals with low sequencing depth (<10,000) after the quality filtering were excluded in the final dataset, hence, in total 229 (172 vaccinated and 57 non-vaccinated controls), 244 (180 vaccinated and 64 non-vaccinated controls), 246 (182 vaccinated and 64 non-vaccinated controls), and 219 (161 vaccinated and 58 non-vaccinated controls) samples were retained at 0, 21, 35, and 118 dpv, respectively.

### 2.6. Biostatistical Analyses

Biostatistical analyses were conducted as described previously [22] with slight modifications. Briefly, alpha diversity was determined using five different estimators: The number of observed OTUs, the abundance-based coverage estimator (ACE), the Chao1 estimator, and Shannon and Simpson indices. ANOVA and Tukey’s honest significant difference (HSD) tests were used to assess the differences among groups. Between-sample diversity (beta diversity) was assessed using the unweighted and weighted UniFrac distance metrics [30], and Principal-coordinate analysis (PCoA) was used to visualize these distances using EMPeror [31]. Overall differences between groups in both weighted and unweighted Unifrac distances were compared using Anosim in QIIME, whereas distances to centroid were calculated using the “betadisper” function of the vegan R package.

The sparse partial least squares (sPLS) regression implemented in ‘mixOmics’ R package (v 6.3.1) [32,33,34] was used for the multivariate analysis of the microbiota data at OTU level in order to identify OTUs that were more predictive of the observed immune response, using all the animals that were subsequently vaccinated at 0 dpv (*n* = 172).

Differential abundance at OTU level between High and Low responder pigs, and between vaccinated and non-vaccinated pigs at each dpv was determined using R package MetagenomeSeq (v 1.20. 1) [35]. In this context, low abundant OTUs were filtered to only include OTUs that were present in at least 20% of the samples at each time point, whereas the effect of gender, batch, and weaning age were included in the model as cofactors. In addition, the list of differentially abundant OTUs between the High and Low responder pigs was subjected to the regularized canonical correlation analysis (rCCA) implemented in the mixOmics (v 6.3.1) R package in order to highlight correlations between the OTUs and anti-*M. hyo* IgG titer levels determined at different time points post-vaccination.

Linear discriminant analysis effect size (LEfSe), an algorithm that focuses on statistical significance and biological consistency [36] was used to identify genera that most likely explain the differences between the High and Low responder groups, as well as between vaccinated and non-vaccinated pigs at different dpv. In this context, genera that were relatively more abundant in a particular sample group were identified by LEfSe using the Kruskal-Wallis test (*p* < 0.05) and the effect size of each of these genera was estimated using linear discriminant analysis (LDA). An LDA score (log_10_) of 2.0 was used as the cut-off for identifying differentially abundant genera. Similarly, multivariate analysis by linear models (MaAsLin) was used to find associations between the microbial abundance and different groups (High and Low responder pigs, vaccinated and non-vaccinated pigs at each dpv, as well as different sampling time points), as described previously [37]. MaAsLin allows the detection of an effect of a metadata/phenotype while deconfounding the effects of any other metadata captured in the study.

### 2.7. Data Availability

Raw sequence reads of the 16S rRNA gene amplicon data are available through the SRA with accession number PRJNA634365.

## 3. Results

### 3.1. Longitudinal Investigation of Swine Fecal Microbiota Confirms Compositional Differences at Different Ages

**Figure 2:**
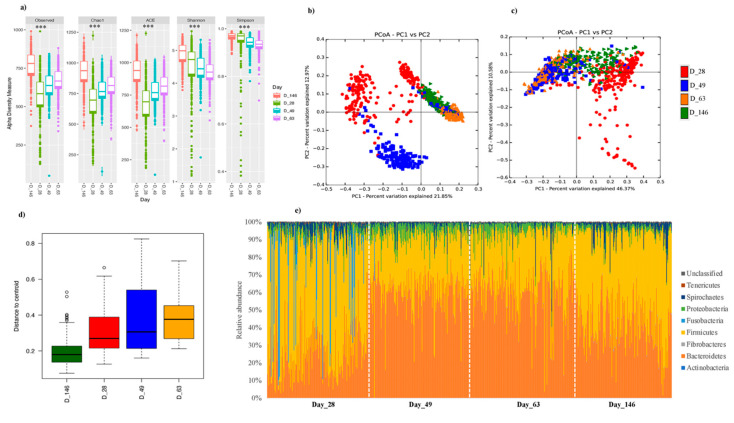

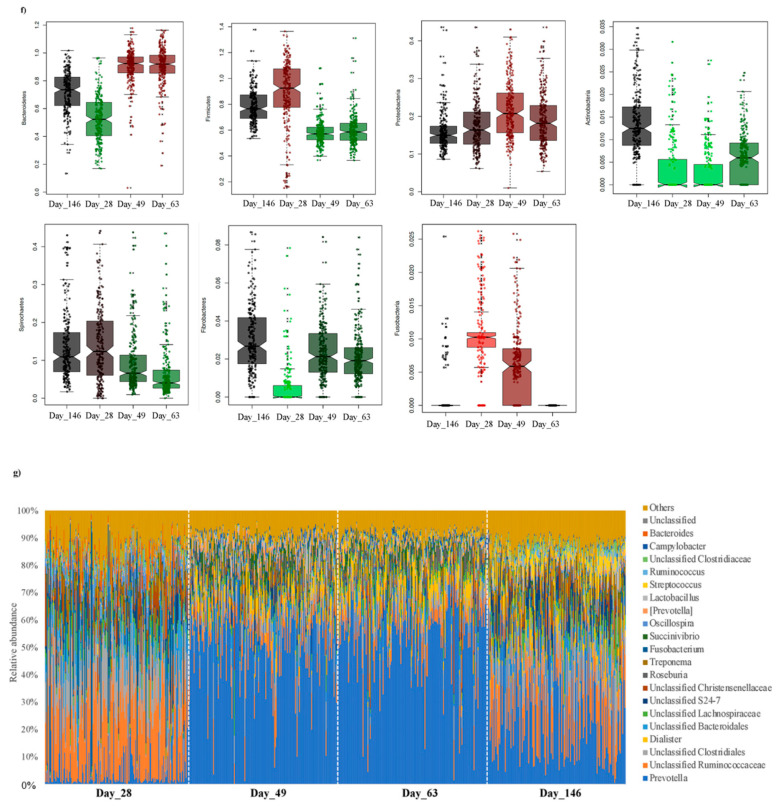
Longitudinal changes in microbial diversity and relative abundance. (**a**) Alpha diversity indices, comparing different sampling dates. ANOVA and Tukey’s honest tests were used to assess the differences *** *p* < 0.0001; see Table S1*)*, (**b**) Unweighted UniFrac distance (Anosim *p* = 0.001), (**c**) Weighted UniFrac distance (Anosim *p* = 0.001), (**d**) Box plot representing the values of the distances to centroid for each sampling date, (**e**) Relative abundance at phylum level as visualized in bar graphs across different sampling time points, (**f**) Associations between bacterial abundance at phylum level and time as analyzed using MaAsLin (only the statistically significant ones are shown). For each phylum, different colors shows the points that are significantly different based on the corrected *p* values (*q* < 0.05), (**g**) Top 20 genera present in all pigs over the course of time (Day_28, Day_49, Day_63, and Day_146 = Postnatal day 28, 49, 63, and 146, respectively). Unclassified = bacteria that were not assigned to any taxonomic classification, others = All other genera that were not part of the top 20.

We analyzed the microbiota composition of all vaccinated and non-vaccinated pigs between different growth stages (PND 28, 49, 63 and 146 corresponding to 0, 21, 35, and 118 dpv). Observed OTUs, Chao1 and ACE revealed a significant progressive increase in richness at each sampling time point (*p* < 0.05; *p* adj < 0.05), whereas Shannon and Simpson revealed a higher diversity at day 146, just before slaughter (*p* < 0.05), but the other sampling time points were not significantly different from each other (*p* adj > 0.05) (Figure 2a; Table S2). Analysis of other variables showed that in some instances, the richness and diversity were significantly influenced by the batch and there was a strong interaction between the sampling time point (Day) and litter (Table S2).

Beta-diversity analysis for both unweighted and weighted Unifrac distances as analyzed using Adonis in QIIME revealed significant differences in microbiota structure between different time points, which suggested that the differences observed were driven by both the abundant and less abundant bacteria (Figure 2b,c). Also, distances to centroid were calculated using the “betadisper” function of the vegan R package, which revealed that the Beta diversity at PND 146 was lower when compared to the other time points, suggesting that the global microbiota composition at this time point was more similar for all animals (Figure 2d).

Phylum Bacteroidetes, Firmicutes, Proteobacteria and Spirochaetes were the predominant phyla present at each time point and accounted for over 95% of all the pig microbial communities in the feces (Figure 2e). MaAsLin analysis revealed significant differences in the abundance of most of the phyla between different sampling time points (Figure 2f). Similarly, MaAsLin analysis at genus level showed significant variations between different time points (Figure S1). Figure 2g shows the relative abundance of the top 20 genera (classified and unclassified) present at different time points. 

### 3.2. Fecal Microbiota Composition of the Vaccinated and Control Pigs Did Not Significantly Differ Post-Vaccination

**Figure 3:**
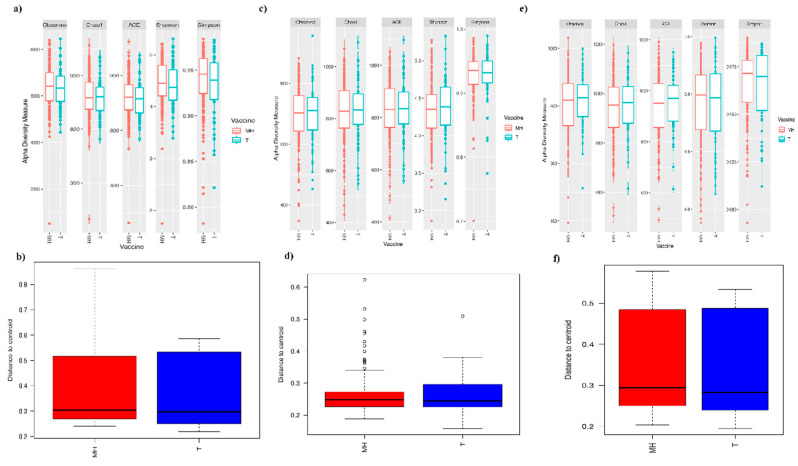
Diversity analysis between the vaccinated and non-vaccinated pigs at three post-vaccination time points; 21, 35, and 118 dpv. (**a**) Alpha diversity indices, comparing vaccinated (MH) vs. non-vaccinated (T) pigs at 21 dpv, (**b**) Box plot representing the values of the distances to centroid for the vaccinated (MH) and non-vaccinated (T) pigs at 21 dpv; (**c**) Alpha diversity indices, comparing vaccinated (MH) vs. non-vaccinated (T) pigs at 35 dpv; (**d**) Box plot representing the values of the distances to centroid for the vaccinated (MH) vs. non-vaccinated (T) pigs at 35 dpv; (**e**) Alpha diversity indices, comparing vaccinated (MH) vs. non-vaccinated (T) pigs at 118 dpv; (**f**) Box plot representing the values of the distances to centroid for the vaccinated (MH) vs. non-vaccinated (T) pigs at 118 dpv. For the alpha diversity, ANOVA and Tukey’s honest tests were used to assess the differences. Distances to centroid were calculated using the “betadisper” function of the vegan R package. No significant differences observed.

We analyzed microbiota composition between the vaccinated and non-vaccinated pigs at three time points post-vaccination (21 dpv, which corresponds to the early response, 35 dpv corresponding to the maximum intensity response, and 118 dpv corresponding to the persistence of response). Diversity analysis showed no significant (*p* > 0.05) differences between the vaccinated and non-vaccinated pigs at each of these time points; however, some alpha diversity indices were significantly different for other factors (Batch and Litter at 21 dpv; Batch, Weaning age, and Litter at 35 dpv; Batch, Litter, Sex, and Weaning age at 118 dpv; Figure 3a–f; Table S3). No other significant differences were observed between the vaccinated and non-vaccinated pigs.

### 3.3. Early-Life Fecal Microbiota Composition before Vaccination as a Predictor of M. hyo Vaccine Response

#### 3.3.1. sPLS Prediction Analysis

**Figure 4:**
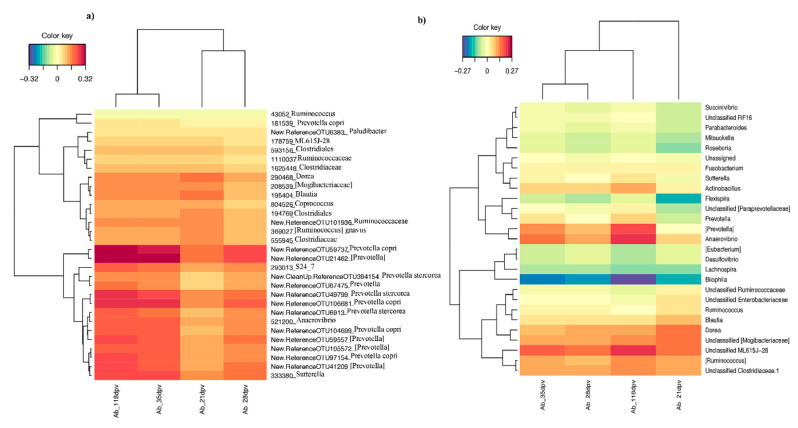
Unsupervised analysis and pre-vaccination feature selection with sparse partial least squares (sPLS) at 0 dpv based on the first two components. (**a**) Clustered Image Map showing the association between the sPLS-selected operational taxonomic units (OTUs) and antibody titers determined at 21, 28, 35, and 118 dpv; (**b**) Clustered image map showing the association between the sPLS-selected taxa at genus level and antibody titers determined at 21, 28, 35, and 118 dpv. The dark brown color shows a positive association, whereas a dark blue color shows a negative association. Ab; antibody titers. Dpv; days post-vaccination.

Data from the pigs that were subsequently vaccinated with *M. hyo* were subjected to sPLS analysis (0 dpv; 172 pigs). In this context, the microbiota data were integrated with antibody titers measured at different time points post-vaccination (Ab_21dpv, Ab_28dpv, Ab_35dpv, and Ab_118dpv), in order to select pre-vaccination OTUs and genera that best-predict the level of antibody response. Several OTUs and genera were selected, and the results are presented using cluster image maps based on the first two components (Figure 4a,b). The results showed that several OTUs annotated principally to *Prevotella copri*, *Prevotella stercorea*, *Prevotella*, [*Prevotella*], *Anaerovibrio* and *Sutterella*, were relatively dominant and could positively predict antibody levels at post-vaccination time points. Other OTUs that showed similar associations with post-vaccination antibody response to a lesser extent included OTUs annotated to S24-7, Clostridieacea, Clostridiales, Ruminococcaceae, *[Ruminococcus] gnavas*, *Coprococcus*, *Blautia*, *Dorea* ML615J-28 and [Mogibacteriaceae]. A visualization of the first three components showed that increased abundance of most OTUs belonging to Ruminococcaceae could be predictors of lower antibody titers, especially at 21 and 35 dpv (Figure S2).

At the genus level, a similar analysis revealed that genera *Anaerovibrio*, [*Prevotella*], *Blautia*, *Dorea*, [*Ruminococcus*], *Ruminococcus*, *Sutterella*, *Prevotella*, unclassified [Mogibacteriaceae], unclassified ML615J-28, and unclassified Clostridiaceae, could positively predict antibody titers, while mostly increased abundance of genera *Bilophila* could predict lower antibody titers. Other genera that would predict lower antibody titers included: *Lachnospira*, *Mitsuokella*, *Desulfovibrio*, *Flexispira*, *Roseburia*, *Lachnospira*, and [*Eubacterium*].

#### 3.3.2. Differential Abundance Analysis at Different Taxonomic Levels

**Figure 5:**
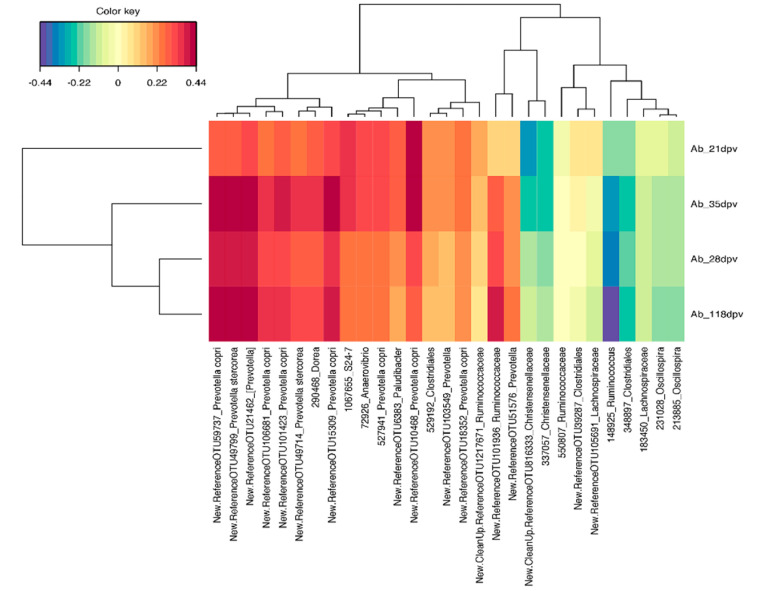
Clustered image map showing the correlation between post-vaccination antibody titers with the pre-vaccination differentially abundant OTUs determined using the 35 dpv classification.

The groups of animals with contrasting responses to vaccination (High vs. Low responders classified based on antibody titers determined at 21, 28, 35, and 118 dpv) were compared using metagenomeSeq in order to identify pre-vaccination OTUs that were differentially abundant between the two groups (Table 1). OTUs appearing as differentially abundant in at least one time point were further subjected to a regularized canonical correlation analysis (rCCA), and the results revealed that the OTUs that were more abundant in High responder pigs showed a positive correlation with the antibody titers measured at the specific dpv that was used to classify the pigs, and this was also observed with the antibody titers measured at other dpv. Conversely, the OTUs that were abundant in Low responder pigs had an opposite effect (Figure 5; Figure S3). Only a few OTUs were differentially abundant between the High and Low responder pigs classified based on antibody titers determined at 21 dpv, and this was expected since this corresponded to an early response, which is not optimal. In this analysis, *Prevotella*, *Coprococcus*, *Ruminococcus* and *Oscillospira* OTUs were more abundant in High responder pigs and also had a positive correlation with antibody titers. Comparison of the groups of High vs. Low responder pigs classified based on antibody titers determined at 28 and 35 dpv, which corresponded to the maximum antibody response intensity, revealed that most of the OTUS that had a positive correlation with antibody titers belonged to *Prevotella*. Contrarily, OTUs belonging to *Oscillospira*, Ruminococcaceae, *Ruminococcus* Clostridiales, and Christensenellaceae had a negative correlation with the antibody titers. A similar trend was observed with the differentially abundant OTUs between High and Low responder pigs classified at 118 dpv.

**Figure 6:**
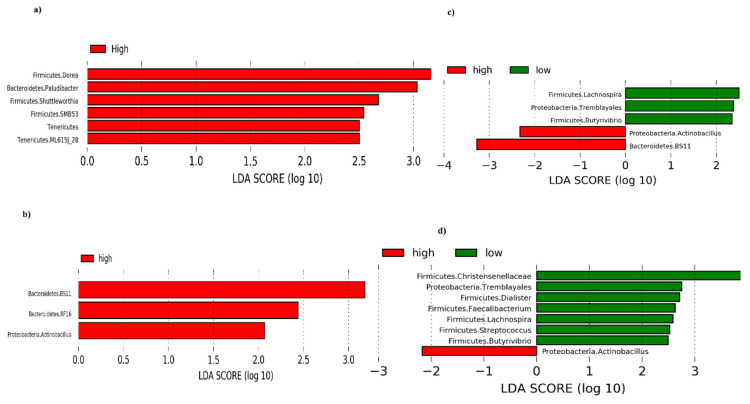
Discriminant analysis of pre-vaccination taxa (at genus level) in High and Low responder pigs selected based on antibody titers determined at different time points. (**a**) Taxa that significantly discriminated between High and Low responder pigs selected based on antibody titers determined at 21 dpv. (**b**) Taxa that significantly discriminated between High and Low responder pigs selected based on antibody titers determined at 28 dpv. (**c**) Taxa that significantly discriminated between High and Low responder pigs selected based on antibody titers determined at 35 dpv. (**d**) Taxa that significantly discriminated between High and Low responder pigs selected based on antibody titers determined at 118 dpv. dpv = days post-vaccination.

The LefSe analysis showed that the High responder pigs classified based on antibody titers determined at 21 dpv were associated with genera *Dorea*, *Paludibacter*, *Shuttleworthia*, *SMB53*, and unclassified Tenericutes, but no significant associations were found with the Low responder pigs (Figure 6a). Similarly, the High responders based on antibody titers at 28 and 35 dpv were associated with genus *Actinobacillus*, unclassified BS11 and RF16, whereas the Low responders were associated with genera *Lachnospira*, *Butyrivibrio* and unclassified Tremblayales (Figure 6b,c). Comparable results were observed in High and Low responder pigs classified based on antibody titers determined at 118 dpv in which genus *Actinobacillus* was associated with the High responder pigs, whereas genera *Butyrivibrio*, *Streptococcus*, *Lachnospira*, *Faecalibacterium*, *Dialister* and unclassified members of Tremblayales and Christensenellaceae, were associated with the Low responder pigs (Figure 6d).

### 3.4. Contrasted Antibody Response Was Not Accompanied by Significant Changes in Microbiota Composition at 21, 35, and 118 dpv

No significant differences were observed between the High and Low responder pigs at 21, 35, and 118 dpv except, we found eight OTUs that were differentially abundant between the two groups at 21 dpv. Four of the differentially abundant OTUs were annotated to *Prevotella*, three were annotated to *Treponema*, and one was annotated to unclassified Bacteroidales. Of the eight OTUs, only one OTU annotated to *Prevotella* was abundant in the group of pigs classified as High, whereas the rest were abundant in the Low responder pigs. No other significant differences were observed.

## 4. Discussion

The gut microbiota plays an important role in shaping mucosal and systemic immunity and protecting the host from pathogen expansion and invasion [5,6]. In this study, we used *M. hyopneumoniae* vaccination to study the potential of early gut microbiota at weaning in predicting future vaccine response, and to explore the dynamics of gut microbiota composition following vaccination. Previous studies in both human and mouse models have demonstrated the importance of commensal bacteria in immune responsiveness to infections in various ways, and to vaccines, such as influenza, polio, and cholera toxins [10,17,38,39]. The studies also suggested that colonization by specific bacteria and reduced dysbiosis in early infancy may improve vaccine responses later in life, whereas other bacteria may lower vaccine response [18,40].

The current study revealed that OTUs annotated to *Prevotella*, [*Prevotella*], *Anaerovibrio*, and *Sutterella*, best-predicted antibody levels at later time points post-vaccination, an observation that was also reflected at the genus level. However, as observed in our previous study [22], the current results showed that differences in vaccination response did not translate to global changes in the composition and diversity of the microbial community, and bacterial diversity indexes alone were not predictive of vaccine response. This is in agreement with a previous study that reported a lack of fecal microbial differences between seroconverters and non-seroconverters in stool samples of 2 month old children analyzed prior to vaccination with rotavirus vaccine [41], and other similar studies, investigating the same vaccine, that reported minimal differences in the gut microbiome between seroconverters and non-seroconverter infants [19,42].

The association of OTUs belonging to *Prevotella* and a few other bacteria with antibody response observed in this study, as well as in our previous study [22], underscores the potential role of members of this genus in swine health. In support of this, a recent study investigating the influence of the intestinal microbiota on colonization and resistance to *Salmonella* and the shedding pattern of naturally exposed pigs reported that among other bacteria, *Prevotella* was more abundant in non-infected pigs, whereas *Anaerovibrio* was more abundant in non-shedder pigs [43]. Similarly, a drastic decrease in the abundance of *Prevotella* among other bacteria was associated with increased *Salmonella* shedding post-infection [44], whereas low lesion score in piglets challenged with the pathogen *M. hyo* has been associated with increased abundance of short chain fatty acid-producing taxa, such as *Prevotella* among others [45].

*Prevotella* is one of the most prevalent genera in the swine gastrointestinal tract, particularly after weaning, and it is widely known for its fiber degradation properties. While it is generally viewed as an indicator of a healthy microbiome in animals, its role in human health studies is quite controversial [46,47]. In this context, *Prevotella* has been shown to positively associate with the production of health-promoting compounds, such as short-chain fatty acids [48,49], influenza-specific IgA titers post-vaccination [39], function in vivo as an immunological adjuvant [50], improve glucose metabolism [51,52], or exhibit overall anti-inflammatory effects [53,54]. Nevertheless, other studies have also reported an association of *Prevotella* with inflammatory phenomenon [55,56,57,58,59,60], mucus layer degradation [59], metabolic syndrome, insulin resistance, as well as glucose intolerance [61]. This can be partly explained by high levels of genomic diversity within *Prevotella* strains of the same species, a condition that is influenced by diet [62,63,64], making it difficult to make a concrete conclusion regarding the involvement of *Prevotella* in vaccine response without the specific strain details. Therefore, strain-level dissection of gut metagenome coupled with an investigation of the metabolites produced by these bacteria may help to explain the precise functional role of *Prevotella* and other taxa in swine health, since it is also unclear whether the effects are due to the specific bacteria themselves or some of their products. This is important because we observed some OTUs belonging to *Prevotella* as more abundant in Low responders and comparable inconclusive results were also reported in the study of *Salmonella* shedding by Arguello and colleagues [43]. Other bacteria of interest, and that warrant further investigation, include genera *Anaerovibrio*, *Sutterella*, *Actinobacillus* and unclassified S24-7, which showed a similar trend exhibited by *Prevotella* in vaccine response. Also, genera *Lachnospira*, *Oscillospira*, and *Butyrivibrio* appeared to have a negative association with the vaccine response, an observation that needs to be studied further. The study of Arguello [43] also reported an increased abundance of *Oscillospira* in *Salmonella* infected pigs.

The microbiota composition of the High and Low responder pigs did not differ post-vaccination except for a few OTUs that were differentially abundant at 21 dpv, which may suggest an early and transient effect and could also be due to the fact that the microbiota was still undergoing compositional changes following weaning. The lack of significant differences between the two groups at later time points post-vaccination may suggest that only the pre-vaccination microbiota was able to influence the vaccine response, and further proposes the presence of an interventional window early in life with dietary, probiotic or other modulatory regimens. This hypothesis agrees with a previous study that reported that antibiotic exposure in infant mice impaired antibody responses to five vaccines that are administered to human infants worldwide, as opposed to antibiotic-treated adult mice that exhibited normal antibody responses to vaccination [20]. Also, we did not find any differences between the vaccinated and the non-vaccinated pigs post-vaccination, which suggests a lack of impact of the vaccine on gut microbiota. This could be attributed to the specific vaccine used in this study or to the changes in the composition of microbiota with time, which might mask the vaccine effect on gut microbiota. However, since we only investigated fecal microbiota, which only represents the condition in the distal part of the gastrointestinal tract, this might not be the situation in the upper gastrointestinal tract (GIT).

The differences in bacterial composition and abundance of specific taxa observed between different growth stages are expected, and have been widely reported [43,65,66,67,68,69]. This is driven majorly by dietary changes, housing, handling, as well as other factors, not forgetting the aspect of microbial maturation with time. In this context, the introduction of solid feed (at weaning) appeared to have a greater overall impact on bacterial community structure and composition with a marked increase in members of phylum Bacteroidetes (particularly *Prevotella*), an observation that has been reported in the past [26,65,66,67,68,69,70]. PCoA sample clustering indicated more inter-individual differences among the PND 28 and 49 samples, which again suggests the influence of diet or other environmental factors. For example, weaning at PND 28 may have played a major role in the composition of the gut microbiota observed at PND 49 as it may take several days for the swine gut microbiome to adapt to a new diet and gut physiology. At the later time points, the gut microbiota was quite stable, which could be an indicator of a more mature microbiome. This was supported by the alpha diversity results that revealed a progressive increase in species richness with time, and higher species richness and evenness at PND 146 compared to the earlier time points.

## 5. Conclusions

The results reported in this study stress the importance of early-life gut microbiota and the need for metagenome sequencing in order to elucidate strain-specific roles, as well as the functional roles of the metabolites, and could be used for future research to define and characterize the composition and function of a ‘healthy’ pig gut microbiota in order to implement disease control strategies. Moreover, considering the concerns of antimicrobial resistance, our results point to a direction where, with further studies, it would be possible to modulate the microbiota of farm animals for a better health-improving vaccine efficiency without the use of antimicrobials. Understanding how the microbial ecosystems evolve at different gastrointestinal sites over time has great biological significance and health implications. However, our study only focused on fecal samples, a strategy that overlooks the spatial microbial dynamics in different intestinal segments. In this context, the role that the mucosa-associated microbiota in different intestinal sections may play in vaccine response remains unknown. Therefore, although the current results mirror those of our previous study, future studies could use the same vaccine in pigs from a different genetic background, production system and country. Further follow up studies are necessary in order to overcome the limitation that only fecal samples were investigated in these studies. Prospective studies are also needed to include information on how the sow (dam) microbiota relates to offspring early-life microbiota composition and subsequent response to vaccination.

## Figures and Tables

**Table 1:** Differentially abundant (DA) OTUs between groups of pigs showing contrasted responses (High vs. Low response) to *M. hyo* vaccination. dpv; days post vaccination.

Differentially Abundant (DA) OTUs					
Ab Response Time Course	dpv	Classification	Number of DA OTUs	Higher Abundance in “High Responders”	Higher Abundance in “Low Responders”
Early (before Booster Vaccination)	21	high vs. low	14	New.ReferenceOTU6913_Prevotella stercorea, 1105328_Coprococcus, New.ReferenceOTU41209_[Prevotella], New.ReferenceOTU10520_[Prevotella], 308786_Ruminococcus, 157772_Oscillospira	New.ReferenceOTU25778_S24−7, New.ReferenceOTU21882_Butyricimonas, New.ReferenceOTU17960_Bacteroidales, New.ReferenceOTU10995_Bacteroidales, New.ReferenceOTU101339_Bacteroidales, New.CleanUp.ReferenceOTU1337388_Bacteroidales, 592649_Clostridiales, 335670_Ruminococcaceae
Maximum Intensity (Post Booster Vaccination)	28	high vs. low	44	New.ReferenceOTU89450_Anaerovibrio, New.ReferenceOTU69704_Prevotella copri, New.ReferenceOTU6913_Prevotella stercorea, New.ReferenceOTU67475_Prevotella, New.ReferenceOTU6383_Paludibacter, New.ReferenceOTU59737_Prevotella copri, New.ReferenceOTU57668_unclassified, New.ReferenceOTU41209_[Prevotella], New.ReferenceOTU21462_[Prevotella], New.ReferenceOTU15309_Prevotella copri, New.ReferenceOTU11599_Prevotella copri, New.ReferenceOTU110597_Prevotella stercorea, New.ReferenceOTU108770_[Prevotella], New.ReferenceOTU106681_Prevotella copri, New.ReferenceOTU101936_Ruminococcaceae, New.ReferenceOTU101423_Prevotella copri, New.ReferenceOTU10106_Prevotella copri, New.ReferenceOTU10063_Anaerovibrio, New.CleanUp.ReferenceOTU891171_Prevotella copri, New.CleanUp.ReferenceOTU816333_Christensenellaceae, New.CleanUp.ReferenceOTU729842_Prevotella copri, 1067655_S24−7, 589329_Prevotella copri, 579750_Lachnospiraceae, 333195_Ruminococcaceae, 301678_Ruminococcaceae, 301012_Clostridiales, 300859_Prevotella, 208949_Bacteroides, 172163_Clostridiales, 72926_Anaerovibrio, 22466_Prevotella	New.ReferenceOTU95104_Lachnospiraceae, New.ReferenceOTU25356_Clostridiales, 827702_Clostridiales, 772972_Clostridiales, 443620_Oscillospira, 369827_Ruminococcaceae, 346686_Oscillospira, 314639_Clostridiales, 263546_Oscillospira, 231028_Oscillospira, 213885_Oscillospira, 152612_Clostridiales
	35	high vs. low	29	72926_Anaerovibrio, 550807_Ruminococcaceae, New.ReferenceOTU49714_Prevotella stercorea, New.ReferenceOTU49799_Prevotella stercorea, New.ReferenceOTU15309_Prevotella copri, New.ReferenceOTU59737_Prevotella copri, New.ReferenceOTU106681_Prevotella copri, New.ReferenceOTU51576_Prevotella, New.ReferenceOTU103549_Prevotella, New.ReferenceOTU18352_Prevotella copri, New.ReferenceOTU101423_Prevotella copri, New.ReferenceOTU10468_Prevotella copri, 527941_Prevotella copri, 290468_Dorea, New.ReferenceOTU21462_[Prevotella], New.ReferenceOTU6383_Paludibacter, 1067655_S24−7, New.cleanUp.ReferenceOTU1217671_Ruminococcaceae, New.ReferenceOTU101936_Ruminococcaceae, 529192_Clostridiales	New.ReferenceOTU39287_Clostridiales, 231028_Oscillospira, 213885_Oscillospira, 148925_Ruminococcus, 348897_Clostridiales, New.cleanUp.ReferenceOTU816333_Christensenellaceae, 337057_Christensenellaceae, New.ReferenceOTU105691_Lachnospiraceae, 183450_Lachnospiraceae
Persistence (Before Slaughtering)	118	high vs. low	69	New.ReferenceOTU9154_S24−7, New.ReferenceOTU75092_Treponema, New.ReferenceOTU6913_Prevotella stercorea, New.ReferenceOTU67475_Prevotella, New.ReferenceOTU59737_Prevotella copri, New.ReferenceTU63368_Treponema, New.ReferenceOTU57668_Unassigned, New.ReferenceOTU49714_Prevotella stercorea, New.ReferenceOTU39287_Clostridiales, New.ReferenceOTU46053_Christensenellaceae, New.ReferenceOTU21462_[Prevotella], New.ReferenceOTU24010_Prevotella copri, New.ReferenceOTU15309_Prevotella copri, New.ReferenceOTU1235_Bacteroidales, New.ReferenceOTU110599_Prevotella copri, New.ReferenceOTU107301_Prevotella, New.ReferenceOTU106681_Prevotella copri, New.ReferenceOTU106222_Parabacteroides, New.ReferenceOTU10468_Prevotella copri, New.ReferenceOTU101611_Prevotella, New.ReferenceOTU101423_Prevotella copri, New.ReferenceOTU10063_Anaerovibrio, New.CleanUp.ReferenceOTU729842_Prevotella copri, New.CleanUp.ReferenceOTU394154_Prevotella stercorea, 3407052_Bacteroides, 647215_Oscillospira, 584951_Ruminococcaceae, 354599_Treponema, 345114_Fusobacterium, 301678_Ruminococcaceae, 288250_Prevotella, 215963_Clostridium, 172163_Clostridiales	New.ReferenceOTU77656_Bacteroidales, New.ReferenceOTU73263_Bacteroidales, New.ReferenceOTU53970_Christensenellaceae, New.ReferenceOTU51770_Prevotella copri, New.ReferenceOTU46057_Clostridiales, New.ReferenceOTU105562_S24−7, New.ReferenceOTU10341_Bacteroides, New.CleanUp.ReferenceOTU915070_Prevotella copri, New.CleanUp.ReferenceOTU870013_Lachnospiraceae, New.CleanUp.ReferenceOTU133342_Lachnospiracea, 4357657_Clostridiaceae, 1106861_Roseburia, 708680_Roseburia, 621472_Roseburia, 538947_Lachnospiraceae, 531614_Prevotella copri, 354632_Ruminicoccaceae, 519836_Prevotella, 339221_Prevotella copri, 337057_Christensenellaceae, 325842_Ruminococcaceae, 325254_Christensenellaceae, 319659_Clostridiales, 314639_Clostridiales, 312490_Clostridiales, 292242_Ruminococcaceae, 291490_Prevotella, 290253_Oscillospira, 274299_Treponema, 259533_Treponema, 248447_Prevotella, 231028_Oscillospira, 213394_Lachnospiraceae, 211935_Lachnospiraceae, 188079_Ruminococcaceae, 178965_Ruminococcaceae, 148925_Ruminococcus

## Supplementary Materials

The following are available online at https://www.mdpi.com/2076-2607/8/8/1151/s1, Figure S1: Relative abundance at genus level across different sampling time points as analyzed using Multivariate analysis by linear models (MaAsLin); Figure S2: Unsupervised analysis and pre-vaccination feature selection with sparse PLS (sPLS) at 0 dpv based on the first 3(1:3) components, Figure S3: Correlation analysis of pre-vaccination differentially abundant OTUs between High and Low responder pigs, Table S1: Pigs that were selected as High or Low responders together with the S/P levels, at different post-vaccination time points; Table S2: Longitudinal changes in microbial diversity and relative abundance; Influence of different factors (sampling date, batch, litter, vaccination, sex and weaning age) on diversity indices, Table S3: Diversity analysis between the vaccinated and non-vaccinated pigs at three post-vaccination time points; 21, 35, and 118 dpv: Influence of different factors (batch, litter, vaccination, sex and weaning age) on diversity indices.

## References

[1] Hoen A.G., Li J., Moulton L.A., O’Toole G.A., Housman M.L., Koestler D.C., Guill M.F., Moore J.H., Hibberd P.L., Morrison H.G. (2015). Associations between gut microbial colonization in early life and respiratory outcomes in cystic fibrosis. Proc. J. Pediatr..

[2] Dou S., Gadonna-Widehem P., Rome V., Hamoudi D., Rhazi L., Lakhal L., Larcher T., Bahi-Jaber N., Pinon-Quintana A., Guyonvarch A. (2017). Characterisation of Early-Life Fecal Microbiota in Susceptible and Healthy Pigs to Post-Weaning Diarrhoea. PLoS ONE.

[3] Ranucci G., Buccigrossi V., De Freitas M.B., Guarino A., Giannattasio A. (2017). Early-Life Intestine Microbiota and Lung Health in Children. J. Immunol. Res..

[4] Fouhse J.M., Yang K., More-Bayona J., Gao Y., Goruk S., Plastow G., Field C.J., Barreda D.R., Willing B.P. (2019). Neonatal Exposure to Amoxicillin Alters Long-Term Immune Response Despite Transient Effects on Gut-Microbiota in Piglets. Front. Immunol..

[5] Kamada N., Núñez G. (2014). Regulation of the immune system by the resident intestinal bacteria. Gastroenterology.

[6] Buffie C.G., Pamer E.G. (2013). Microbiota-mediated colonization resistance against intestinal pathogens. Nat. Rev. Immunol..

[7] Bauer H., Horowitz R.E., Levenson S.M., Popper H. (1963). The response of the lymphatic tissue to the microbial flora. Studies on germfree mice. Am. J. Pathol..

[8] Lamousé-Smith E.S., Tzeng A., Starnbach M.N. (2011). The intestinal flora is required to support antibody responses to systemic immunization in infant and germ free mice. PLoS ONE.

[9] Zimmermann P., Curtis N. (2018). The influence of the intestinal microbiome on vaccine responses. Vaccine.

[10] Lynn D.J., Pulendran B. (2017). The potential of the microbiota to influence vaccine responses. J. Leukoc. Biol..

[11] Velasquez D.E., Parashar U., Jiang B. (2018). Decreased performance of live attenuated, oral rotavirus vaccines in low-income settings: Causes and contributing factors. Expert Rev. Vaccines.

[12] Valdez Y., Brown E.M., Finlay B.B. (2014). Influence of the microbiota on vaccine effectiveness. Trends Immunol..

[13] Ferreira R.B.R., Antunes L.C.M., Brett Finlay B. (2010). Should the human microbiome be considered when developing vaccines?. PLoS Pathog..

[14] Collins N., Belkaid Y. (2018). Do the microbiota influence vaccines and protective immunity to pathogens?: Engaging our endogenous adjuvants. Cold Spring Harb. Perspect. Biol..

[15] Littman D.R. (2018). Do the microbiota influence vaccines and protective immunity to pathogens?: If so, is there potential for efficacious microbiota-based vaccines?. Cold Spring Harb. Perspect. Biol..

[16] Macpherson A.J. (2018). Do the Microbiota Influence Vaccines and Protective Immunity to Pathogens?. Cold Spring Harb. Perspect. Biol..

[17] Oh J.Z., Ravindran R., Chassaing B., Carvalho F.A., Maddur M.S., Bower M., Hakimpour P., Gill K.P., Nakaya H.I., Yarovinsky F. (2014). TLR5-mediated sensing of gut microbiota is necessary for antibody responses to seasonal influenza vaccination. Immunity.

[18] Huda M.N., Lewis Z., Kalanetra K.M., Rashid M., Ahmad S.M., Raqib R., Qadri F., Underwood M.A., Mills D.A., Stephensen C.B. (2014). Stool microbiota and vaccine responses of infants. Pediatrics.

[19] Harris V.C., Armah G., Fuentes S., Korpela K.E., Parashar U., Victor J.C., Tate J., de Weerth C., Giaquinto C., Wiersinga W.J. (2017). Significant Correlation between the Infant Gut Microbiome and Rotavirus Vaccine Response in Rural Ghana. J. Infect. Dis..

[20] Lynn M.A., Tumes D.J., Choo J.M., Sribnaia A., Blake S.J., Leong L.E.X., Young G.P., Marshall H.S., Wesselingh S.L., Rogers G.B. (2018). Early-Life Antibiotic-Driven Dysbiosis Leads to Dysregulated Vaccine Immune Responses in Mice. Cell Host Microbe.

[21] Yitbarek A., Astill J., Hodgins D.C., Parkinson J., Nagy É., Sharif S. (2019). Commensal gut microbiota can modulate adaptive immune responses in chickens vaccinated with whole inactivated avian influenza virus subtype H9N2. Vaccine.

[22] Munyaka P.M., Kommadath A., Fouhse J., Wilkinson J., Diether N., Stothard P., Estellé J., Rogel-Gaillard C., Plastow G., Willing B.P. (2019). Characterization of whole blood transcriptome and early-life fecal microbiota in high and low responder pigs before, and after vaccination for Mycoplasma hyopneumoniae. Vaccine.

[23] Blanc F., Maroilley T., Revilla M., Lemonnier G., Leplat J., Billon Y., Bouchez O., Bidanel J., Bed’Hom B., Pinard-van der Laan M. (2020). Influence of genetics and pre-vaccination blood transcriptome on the variability of antibody levels after vaccination against Mycoplasma hyopneumoniae in pigs.

[24] GenESI, INRAE, Pig Phenotyping and Innovative Breeding Facility.

[25] Godon J.J., Zumstein E., Dabert P., Habouzit F., Moletta R. (1997). Molecular microbial diversity of an anaerobic digestor as determined by small-subunit rDNA sequence analysis. Appl. Environ. Microbiol..

[26] Massacci F.R., Berri M., Lemonnier G., Guettier E., Blanc F., Jardet D., Rossignol M.N., Mercat M.-J., Doré J., Lepage P. (2020). Late weaning is associated with increased microbial diversity and Faecalibacterium prausnitzii abundance in the fecal microbiota of piglets. Anim. Microbiome.

[27] Caporaso J.G., Kuczynski J., Stombaugh J., Bittinger K., Bushman F.D., Costello E.K., Fierer N., Pẽa A.G., Goodrich J.K., Gordon J.I. (2010). QIIME allows analysis of high-throughput community sequencing data. Nat. Methods.

[28] Crespo-Piazuelo D., Migura-Garcia L., Estellé J., Criado-Mesas L., Revilla M., Castelló A., Muñoz M., García-Casco J.M., Fernández A.I., Ballester M. (2019). Association between the pig genome and its gut microbiota composition. Sci. Rep..

[29] Bokulich N.A., Subramanian S., Faith J.J., Gevers D., Gordon J.I., Knight R., Mills D.A., Caporaso J.G. (2013). Quality-filtering vastly improves diversity estimates from Illumina amplicon sequencing. Nat. Methods.

[30] Lozupone C., Knight R. (2005). UniFrac: A new phylogenetic method for comparing microbial communities. Appl. Environ. Microbiol..

[31] Vázquez-Baeza Y., Pirrung M., Gonzalez A., Knight R. (2013). EMPeror: A tool for visualizing high-throughput microbial community data. Gigascience.

[32] Cao K.A.L., Costello M.E., Lakis V.A., Bartolo F., Chua X.Y., Brazeilles R., Rondeau P. (2016). MixMC: A multivariate statistical framework to gain insight into microbial communities. PLoS ONE.

[33] Rohart F., Gautier B., Singh A., Lê Cao K.A. (2017). mixOmics: An R package for ‘omics feature selection and multiple data integration. PLoS Comput. Biol..

[34] Lê Cao K.A., Boitard S., Besse P. (2011). Sparse PLS discriminant analysis: Biologically relevant feature selection and graphical displays for multiclass problems. BMC Bioinformatics.

[35] Paulson J.N., Colin Stine O., Bravo H.C., Pop M. (2013). Differential abundance analysis for microbial marker-gene surveys. Nat. Methods.

[36] Segata N., Izard J., Waldron L., Gevers D., Miropolsky L., Garrett W.S., Huttenhower C. (2011). Metagenomic biomarker discovery and explanation. Genome Biol..

[37] Morgan X.C., Tickle T.L., Sokol H., Gevers D., Devaney K.L., Ward D.V., Reyes J.A., Shah S.A., LeLeiko N., Snapper S.B. (2012). Dysfunction of the intestinal microbiome in inflammatory bowel disease and treatment. Genome Biol..

[38] Hall J.A., Bouladoux N., Sun C.M., Wohlfert E.A., Blank R.B., Zhu Q., Grigg M.E., Berzofsky J.A., Belkaid Y. (2008). Commensal DNA Limits Regulatory T Cell Conversion and Is a Natural Adjuvant of Intestinal Immune Responses. Immunity.

[39] Salk H.M., Simon W.L., Lambert N.D., Kennedy R.B., Grill D.E., Kabat B.F., Poland G.A. (2016). Taxa of the Nasal Microbiome Are Associated with Influenza-Specific IgA Response to Live Attenuated Influenza Vaccine. PLoS ONE.

[40] Huda M., Ahmad S., Kalanetra K., Taft D., Lewis Z., Raqib R., Mills D., Stephensen C. (2017). Infant stool microbiota at the time of vaccination at 6 w of age is associated with vaccine responses measured at 2 y of age. Immunology.

[41] Fix J., Chandrashekhar K., Perez J., Bucardo F., Hudgens M.G., Yuan L., Twitchell E., Azcarate-Peril M.A., Vilchez S., Becker-Dreps S. (2019). Association between Gut Microbiome Composition and Rotavirus Vaccine Response among Nicaraguan Infants. Am. J. Trop. Med. Hyg..

[42] Parker E.P.K., Praharaj I., Zekavati A., Lazarus R.P., Giri S., Operario D.J., Liu J., Houpt E., Iturriza-Gómara M., Kampmann B. (2018). Influence of the intestinal microbiota on the immunogenicity of oral rotavirus vaccine given to infants in south India. Vaccine.

[43] Argüello H., Estellé J., Leonard F.C., Crispie F., Cotter P.D., O’Sullivan O., Lynch H., Walia K., Duffy G., Lawlor P.G. (2019). Influence of the Intestinal Microbiota on Colonization Resistance to Salmonella and the Shedding Pattern of Naturally Exposed Pigs. mSystems.

[44] Bearson S.M.D., Allen H.K., Bearson B.L., Looft T., Brunelle B.W., Kich J.D., Tuggle C.K., Bayles D.O., Alt D., Levine U.Y. (2013). Profiling the gastrointestinal microbiota in response to Salmonella: Low versus high Salmonella shedding in the natural porcine host. Infect. Genet. Evol..

[45] Surendran Nair M., Eucker T., Martinson B., Neubauer A., Victoria J., Nicholson B., Pieters M. (2019). Influence of pig gut microbiota on Mycoplasma hyopneumoniae susceptibility. Vet. Res..

[46] Cani P.D. (2018). Human gut microbiome: Hopes, threats and promises. Gut.

[47] Ley R. (2016). Gut microbiota in 2015: Prevotella in the gut: Choose carefully. Nat Rev Gastroenterol Hepatol..

[48] De Filippis F., Pellegrini N., Laghi L., Gobbetti M., Ercolini D. (2016). Unusual sub-genus associations of faecal Prevotella and Bacteroides with specific dietary patterns. Microbiome.

[49] De Filippo C., Cavalieri D., Di Paola M., Ramazzotti M., Poullet J.B., Massart S., Collini S., Pieraccini G., Lionetti P. (2010). Impact of diet in shaping gut microbiota revealed by a comparative study in children from Europe and rural Africa. Proc. Natl. Acad. Sci. USA.

[50] Chilton P.M., Hadelq D.M., To T.T., Mitchell T.C., Darveau R.P. (2013). Adjuvant activity of naturally occurring monophosphoryl lipopolysaccharide preparations from mucosa-associated bacteria. Infect. Immun..

[51] Kovatcheva-Datchary P., Nilsson A., Akrami R., Lee Y.S., De Vadder F., Arora T., Hallen A., Martens E., Björck I., Bäckhed F. (2015). Dietary Fiber-Induced Improvement in Glucose Metabolism Is Associated with Increased Abundance of Prevotella. Cell Metab..

[52] De Vadder F., Kovatcheva-Datchary P., Zitoun C., Duchampt A., Bäckhed F., Mithieux G. (2016). Microbiota-Produced Succinate Improves Glucose Homeostasis via Intestinal Gluconeogenesis. Cell Metab..

[53] Vitaglione P., Mennella I., Ferracane R., Rivellese A.A., Giacco R., Ercolini D., Gibbons S.M., La Storia A., Gilbert J.A., Jonnalagadda S. (2015). Whole-grain wheat consumption reduces inflammation in a randomized controlled trial on overweight and obese subjects with unhealthy dietary and lifestyle behaviors: Role of polyphenols bound to cereal dietary fiber. Am. J. Clin. Nutr..

[54] Angelis M.D., Montemurno E., Vannini L., Cosola C., Cavallo N., Gozzi G., Maranzano V., Di Cagno R., Gobbetti M., Gesualdo L. (2015). Effect of whole-grain barley on the human fecal microbiota and metabolome. Appl. Environ. Microbiol..

[55] Lozupone C.A., Rhodes M.E., Neff C.P., Fontenot A.P., Campbell T.B., Palmer B.E. (2014). HIV-induced alteration in gut Microbiota: Driving factors, consequences, and effects of antiretroviral therapy. Gut Microbes.

[56] Elinav E., Strowig T., Kau A.L., Henao-Mejia J., Thaiss C.A., Booth C.J., Peaper D.R., Bertin J., Eisenbarth S.C., Gordon J.I. (2011). NLRP6 inflammasome regulates colonic microbial ecology and risk for colitis. Cell.

[57] Scher J.U., Sczesnak A., Longman R.S., Segata N., Ubeda C., Bielski C., Rostron T., Cerundolo V., Pamer E.G., Abramson S.B. (2013). Expansion of intestinal Prevotella copri correlates with enhanced susceptibility to arthritis. Elife.

[58] Maeda Y., Kurakawa T., Umemoto E., Motooka D., Ito Y., Gotoh K., Hirota K., Matsushita M., Furuta Y., Narazaki M. (2016). Dysbiosis Contributes to Arthritis Development via Activation of Autoreactive T Cells in the Intestine. Arthritis Rheumatol..

[59] Rolhion N., Chassaing B., Nahori M.A., de Bodt J., Moura A., Lecuit M., Dussurget O., Bérard M., Marzorati M., Fehlner-Peach H. (2019). A Listeria monocytogenes Bacteriocin Can Target the Commensal Prevotella copri and Modulate Intestinal Infection. Cell Host Microbe.

[60] Alpizar-Rodriguez D., Lesker T.R., Gronow A., Gilbert B., Raemy E., Lamacchia C., Gabay C., Finckh A., Strowig T. (2019). Prevotella copri in individuals at risk for rheumatoid arthritis. Ann. Rheum. Dis..

[61] Pedersen H.K., Gudmundsdottir V., Nielsen H.B., Hyotylainen T., Nielsen T., Jensen B.A.H., Forslund K., Hildebrand F., Prifti E., Falony G. (2016). Human gut microbes impact host serum metabolome and insulin sensitivity. Nature.

[62] Gupta V.K., Chaudhari N.M., Iskepalli S., Dutta C. (2015). Divergences in gene repertoire among the reference Prevotella genomes derived from distinct body sites of human. BMC Genomics.

[63] De Filippis F., Pasolli E., Tett A., Tarallo S., Naccarati A., De Angelis M., Neviani E., Cocolin L., Gobbetti M., Segata N. (2019). Distinct Genetic and Functional Traits of Human Intestinal Prevotella copri Strains Are Associated with Different Habitual Diets. Cell Host Microbe.

[64] Fehlner-Peach H., Magnabosco C., Raghavan V., Scher J.U., Tett A., Cox L.M., Gottsegen C., Watters A., Wiltshire-Gordon J.D., Segata N. (2019). Distinct Polysaccharide Utilization Profiles of Human Intestinal Prevotella copri Isolates. Cell Host Microbe.

[65] Wang X., Tsai T., Deng F., Wei X., Chai J., Knapp J., Apple J., Maxwell C.V., Lee J.A., Li Y. (2019). Longitudinal investigation of the swine gut microbiome from birth to market reveals stage and growth performance associated bacteria. Microbiome.

[66] Liu Y., Zheng Z., Yu L., Wu S., Sun L., Wu S., Xu Q., Cai S., Qin N., Bao W. (2019). Examination of the temporal and spatial dynamics of the gut microbiome in newborn piglets reveals distinct microbial communities in six intestinal segments. Sci. Rep..

[67] De Rodas B., Youmans B.P., Danzeisen J.L., Tran H., Johnson T.J. (2018). Microbiome profiling of commercial pigs from farrow to finish. J. Anim. Sci..

[68] Zhao W., Wang Y., Liu S., Huang J., Zhai Z., He C., Ding J., Wang J., Wang H., Fan W. (2015). The dynamic distribution of porcine microbiota across different ages and gastrointestinal tract segments. PLoS ONE.

[69] Mach N., Berri M., Estellé J., Levenez F., Lemonnier G., Denis C., Leplat J.J., Chevaleyre C., Billon Y., Doré J. (2015). Early-life establishment of the swine gut microbiome and impact on host phenotypes. Environ. Microbiol. Rep..

[70] Guevarra R.B., Lee J.H., Lee S.H., Seok M.J., Kim D.W., Kang B.N., Johnson T.J., Isaacson R.E., Kim H.B. (2019). Piglet gut microbial shifts early in life: Causes and effects. J. Anim. Sci. Biotechnol..

