# Supplementary figures and images for: Discovery of Predictors of Mycoplasma hyopneumoniae Vaccine Response Efficiency in Pigs: 16S rRNA Gene Fecal Microbiota Analysis
Source: Microorganisms. 2020 Jul 29;8(8):1151. doi: 10.3390/microorganisms8081151 (PMC7464067; doi:10.3390/microorganisms8081151)

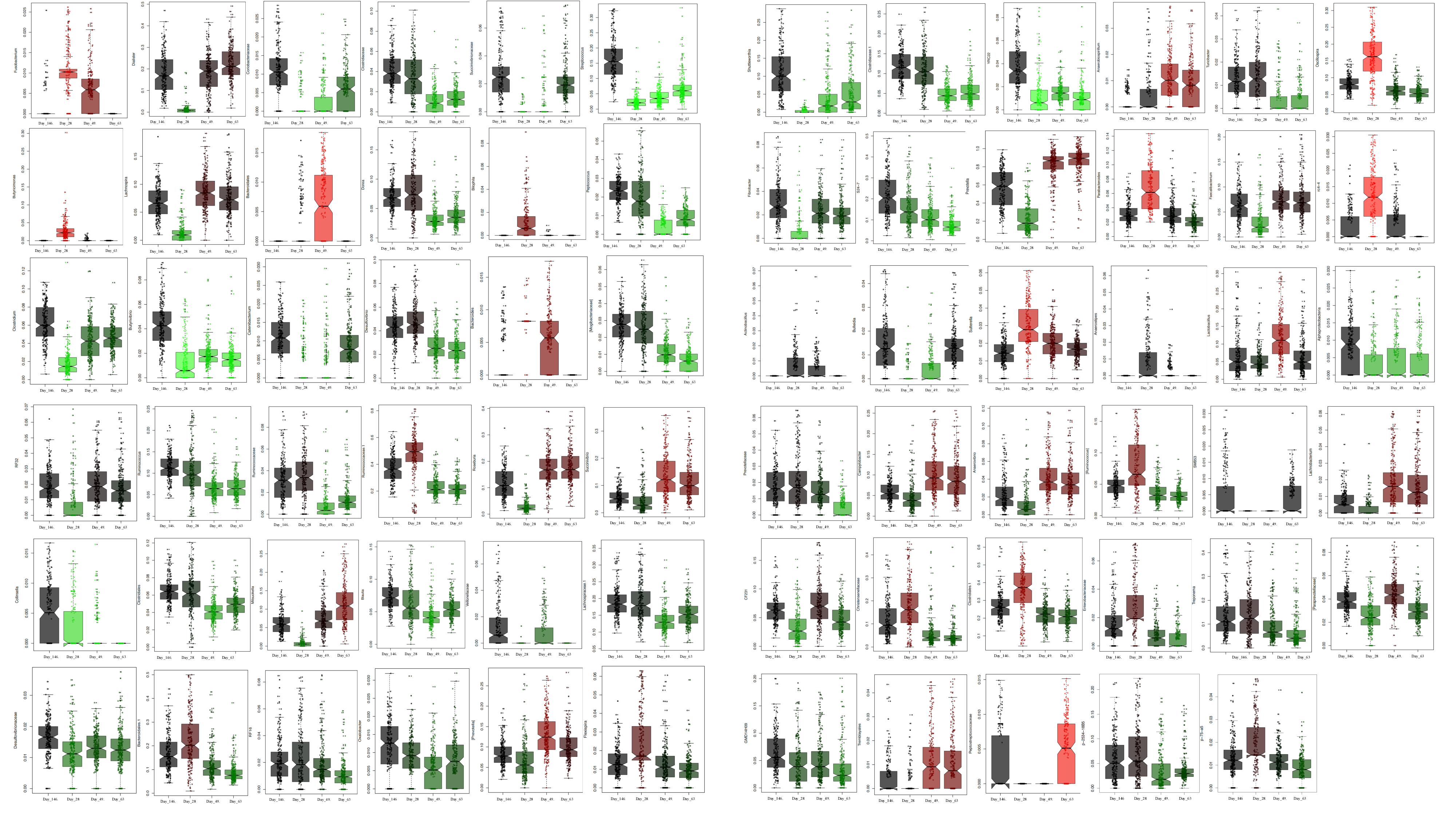

Supplement: Supplementary file 1 [file microorganisms-08-01151-s001.zip › Supplementary_information_revised/Figure S1.tiff]

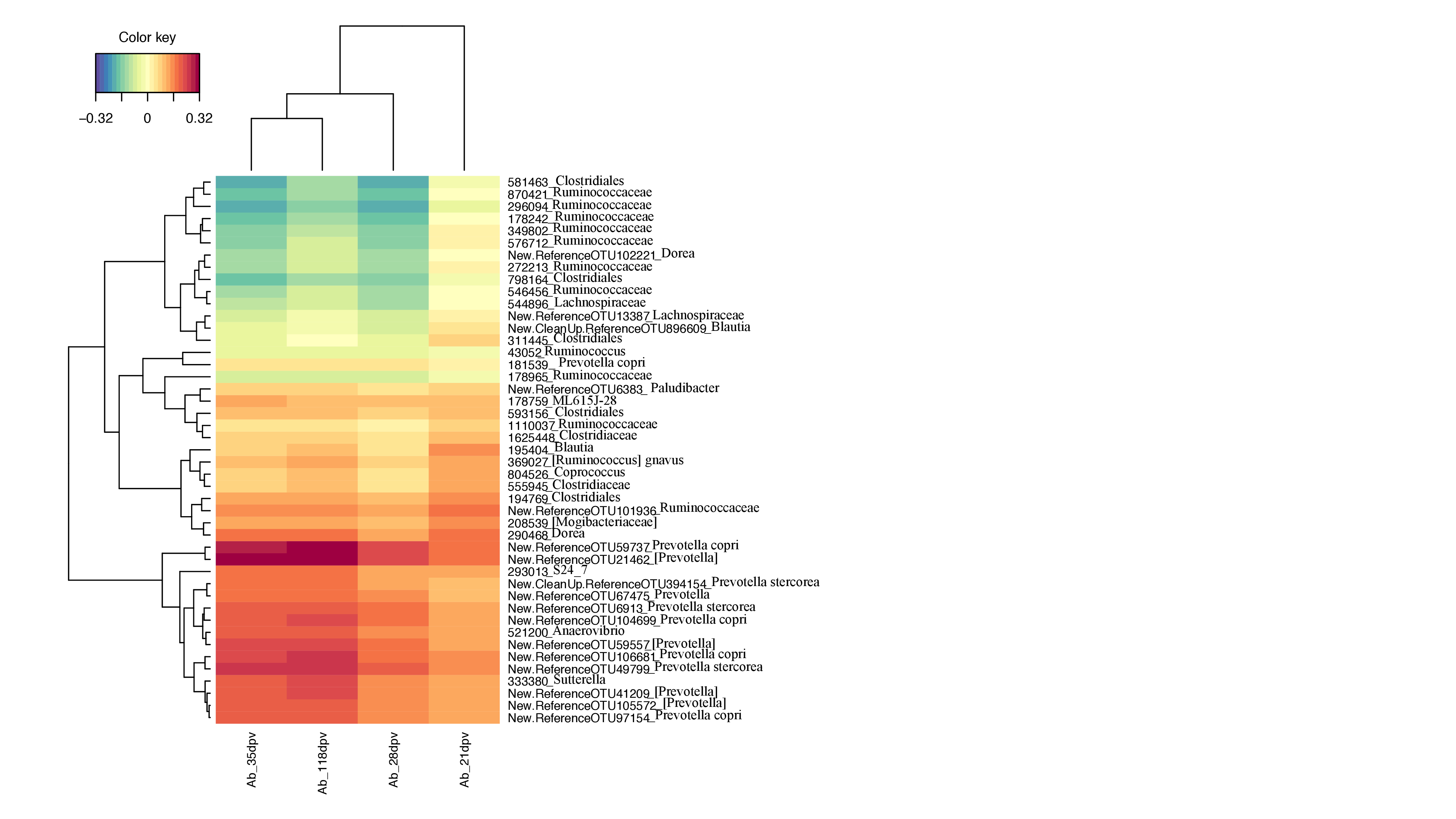

Supplement: Supplementary file 1 [file microorganisms-08-01151-s001.zip › Supplementary_information_revised/Figure S2.tiff]

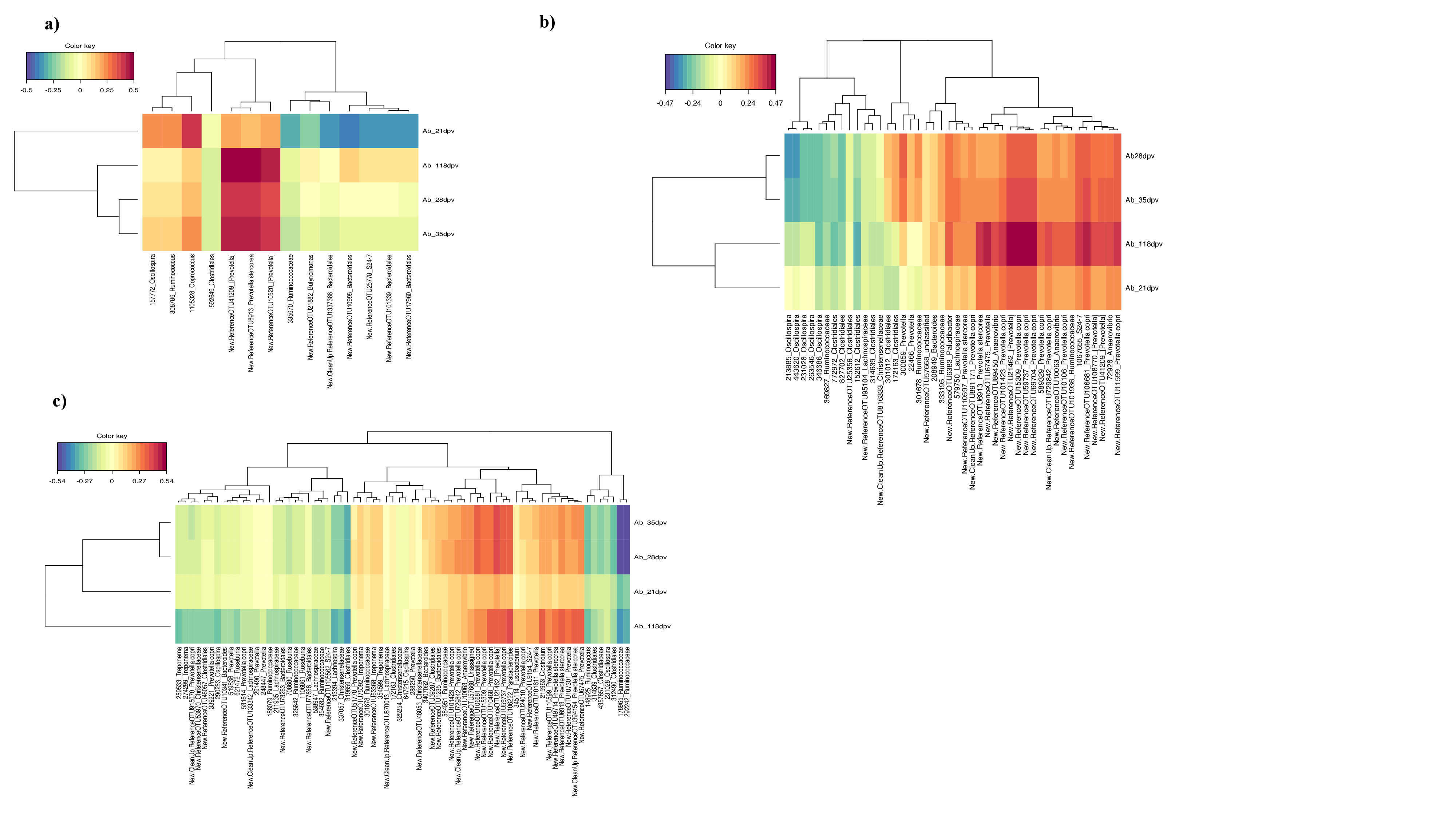

Supplement: Supplementary file 1 [file microorganisms-08-01151-s001.zip › Supplementary_information_revised/Figure S3.tiff]
